# Neural stem cell transplantation in rodent models of traumatic brain injury: a systematic review and meta-analysis

**DOI:** 10.3389/fbioe.2026.1823886

**Published:** 2026-05-21

**Authors:** Yifan Song, Fangzheng Liu, Junling Xu, Wentao Wu, Youchao Xiao, Yanfei Jia, Lu Jin, Ning Qiao, Kefan Cai, Siming Ru, Xin Liu, Jintian Xu, Lei Cao, Songbai Gui

**Affiliations:** 1 Department of Neurosurgery, Beijing Tiantan Hospital, Capital Medical University, Beijing, China; 2 Department of Obstetrics and Gynecology, The Affiliated Hospital of Qingdao University, Qingdao, China; 3 Department of Neurosurgery, The First Affiliated Hospital of Ningbo University, Ningbo, Zhejiang, China; 4 Department of Neurosurgery, Peking University First Hospital, Peking University, Beijing, China

**Keywords:** cell transplantation, lesion volume, meta-analysis, modified neurological severity score, neural stem cells, preclinical studies, rodent models, systematic review

## Abstract

**Background:**

Neural stem cell (NSC) transplantation holds significant promise as a treatment for traumatic brain injury (TBI); however, its therapeutic effects are inconsistent. To address this, we conducted a systematic review and meta-analysis to evaluate the efficacy of NSC transplantation in preclinical studies.

**Methods:**

We searched PubMed, Embase, and Web of Science for studies published up to January 2024 that investigated the effects of NSC transplantation in rodent models of TBI. The primary outcome was the modified Neurological Severity Score (mNSS), and the secondary outcome was lesion volume. Standardized mean differences (SMD) and confidence intervals (CI) were calculated using a random-effects model. The quality of the included studies was assessed using the Collaborative Approach to Meta-Analysis and Review of Animal Data from Experimental Studies (CAMARADES) checklist. All statistical analyses were performed using Stata 17.0.

**Results:**

A total of 18 preclinical studies met the inclusion criteria, reporting 11 outcomes associated with the mNSS and 18 outcomes associated with lesion volume. The quality assessment based on the CAMARADES checklist yielded a median score of 7.00 (interquartile range: 6.25-7.00). The meta-analysis revealed that NSC transplantation significantly reduced the mNSS (SMD = -0.96; 95% CI: -1.40 to -0.51) and lesion volume (SMD = -0.99; 95% CI: -1.46 to -0.52) in rodent models of TBI. Significant heterogeneity was observed in the results for lesion volume. Transplantation dose and timing may be potential sources of this heterogeneity.

**Conclusion:**

Our systematic review and meta-analysis indicate that NSC transplantation can improve neurological function and reduce lesion volume in rodent models of TBI.

**Systematic Review Registration:**

https://www.crd.york.ac.uk/PROSPERO/view/CRD42024505552, identifier CRD42024505552.

## 1 Introduction

Traumatic brain injury (TBI) remains a significant public health concern, contributing to substantial socio-economic burdens. Research estimates that approximately 69 million individuals experience TBI annually, with this number on the rise (Dams-O’Connor et al., 2023; Dewan et al., 2019). Among younger populations, road traffic accidents are the leading cause of these injuries, while falls are more prevalent among older adults. Moreover, the likelihood of experiencing TBI is approximately twice as high in males compared to females (Jiang et al., 2019). According to the classic Glasgow Coma Scale, TBIs are classified as mild (a score of 13–15), moderate (a score of 9–12), or severe (a score of 8 or less) (Najem et al., 2018; Maas et al., 2022). TBI involves primary brain injuries that occur directly at the time of the incident, including skull fractures, contusions, lacerations, and cerebral hemorrhage. Secondary injuries, which develop following the initial trauma, are primarily due to cerebral edema, ischemia, inflammatory responses, and oxidative stress (Ghajar, 2000; Kochanek et al., 2013).

Currently, mild TBIs are primarily managed symptomatically. In contrast, treatment options for moderate to severe injuries vary depending on the condition and include surgical decompression and the maintenance of cerebral perfusion pressure (Jha et al., 2019; Feng and Jiang, 2020). However, these treatments have a limited ability to restore lost neurological functions. Therefore, several novel treatment modalities, including stem cell therapy, have been proposed to repair these lost neurological functions (Zhou et al., 2019).

Stem cells, which are capable of self-renewal and differentiation into various cell types, include embryonic stem cells, hematopoietic stem cells, mesenchymal stem cells (MSCs), neural stem cells, and others (Trounson and McDonald, 2015; Li et al., 2024). Among these, neural stem cells hold significant potential for treating various neurological disorders owing to their differentiation properties. Unlike MSCs, which are primarily thought to act through paracrine signaling and immunomodulation, NSCs are lineage-committed neural progenitors with the capacity to differentiate into neurons, astrocytes, and oligodendrocytes, thereby providing a stronger basis for cell replacement, circuit reconstruction, and remyelination in the injured brain (Kaminska et al., 2022). In addition, NSCs can secrete multiple neurotrophic factors (such as BDNF, GDNF, NGF), modulate neuroinflammation, and interact with the host microenvironment, suggesting that they may combine regenerative and immunoregulatory effects after TBI (Wang et al., 2024; Guo et al., 2022). Preclinical studies have explored the efficacy of neural stem cell treatments for TBIs. Sullivan et al. found that NSCs transplanted into the lateral ventricle of mice with TBI significantly reduce neuroinflammation and decrease the activation of glial cells and macrophages in the corpus callosum (Sullivan and Armstrong, 2017). Additionally, Ji et al. demonstrated that transplanting NSCs overexpressing Rimkla regulates the glutamate cycle, thereby promoting synaptic repair and cognitive recovery post-injury in mice (Ji et al., 2023). However, sufficient evidence supporting their effectiveness as a clinical treatment modality remains lacking.

Given their cost-effectiveness and biological similarities to humans, rodent models are predominantly used in preclinical TBI research. Efficacy of interventions is typically evaluated by assessing neurological function and measuring lesion volume. The modified Neurological Severity Score (mNSS) is widely accepted and serves as the primary tool for assessing neurological function by most researchers (Wu et al., 2020; Xu et al., 2021; Liu et al., 2023). Conversely, lesion volume provides a direct measure of the effectiveness of brain tissue repair at the injury site (Luo et al., 2024; Cheng et al., 2023).

Previous meta-analyses have evaluated stem cell-based therapies for TBI and other neurological injury models, including MSCs and stem cell-derived extracellular vesicles. However, no prior meta-analysis has specifically focused on NSC transplantation in rodent models of TBI. Given the distinct neural lineage properties and regenerative potential of NSCs, a dedicated quantitative synthesis is warranted. Therefore, in this study, we collected and screened relevant preclinical studies from various databases to conduct a meta-analysis, assessing the therapeutic effects of NSC transplantation on the mNSS and lesion volume in these models.

## 2 Methods

This systematic review and meta-analysis has been reported in line with PRISMA (Preferred Reporting Items for Systematic Reviews and Meta-Analyses) and AMSTAR (Assessing the methodological quality of systematic reviews) Guidelines (Page et al., 2021a; Shea et al., 2017; Page et al., 2021b). The protocol was prospectively registered in the International Prospective Register of Systematic Reviews (PROSPERO) (ID: CRD42024505552).

### 2.1 Search strategy

An online search was performed in three databases—PubMed, Embase, and Web of Science—to identify eligible studies. The search terms used were as follows: (“Neural Stem Cells” OR “Cell, Neural Stem” OR “Cells, Neural Stem” OR “Neural Stem Cell” OR “Stem Cell, Neural” OR “Stem Cells, Neural”) AND (“Traumatic Brain Injury” OR “Brain Injuries, Traumatic” OR “Traumatic Brain Injuries” OR “Trauma, Brain” OR “Brain Trauma” OR “Brain Traumas” OR “Traumas, Brain” OR “TBI (Traumatic Brain Injury)” OR “Encephalopathy, Traumatic” OR “Encephalopathies, Traumatic” OR “Traumatic Encephalopathies” OR “Injury, Brain, Traumatic” OR “Traumatic Encephalopathy” OR “TBIs (Traumatic Brain Injuries)” OR “TBI (Traumatic Brain Injuries).” Only studies published in English were included. The detailed search strategy is provided in Supplementary Material S1.

### 2.2 Inclusion and exclusion criteria

Based on the Population, Intervention, Control, Outcome, and Study design (PICOS) scheme, the inclusion criteria were established as follows: (1) the subjects were rodents, specifically rats and mice; (2) a preclinical TBI model was induced; (3) the intervention group was treated with NSCs; (4) studies including a separate control group (saline, culture medium, or no treatment); (5) studies providing adequate information regarding the mNSS or lesion volume; (6) original research studies; (7) and studies published in English.

The exclusion criteria were as follows: (1) purely *in vitro* studies; (2) studies involving modified NSCs or NSCs combined with other materials or drugs; (3) studies that did not provide precise outcome data; (4) studies lacking clear quantification of the number of animals in both experimental and control groups; and (5) literature reviews, book chapters, letters, expert opinions, conference abstracts, or editorial correspondence that did not include original data.

### 2.3 Study selection

Retrieved articles were managed using EndNote X9. Prior to conducting any literature research, we imported the data into the software. Subsequently, duplicate articles were identified and removed. Two authors independently reviewed the studies based on the inclusion and exclusion criteria. Screening was performed in two phases to determine the final studies to be included in the review: (1) an initial screening based on titles and abstracts, and (2) a full-text screening of eligible articles for final inclusion. In each phase, two authors independently assessed each article. Any discrepancies were resolved through discussion or by consulting a third author.

### 2.4 Data extraction

The following information was extracted from each study: first author, year of publication, country, animal species, sex and weight of the animals, lesion models, source of NSCs, transplantation doses, transplantation route, transplantation timing, duration, and outcomes of the mNSS as well as lesion volume. Data were extracted from the included studies by two independent researchers, which will be carefully compared with each other. Any discrepancies were resolved by consulting a third consultant. When only graphs were available, the data values were derived using the GetData Graph Digitizer software (version 2.26). If data remained missing or unclear, we attempted to contact the authors for clarification. However, if these efforts failed, we excluded these data from our analysis. For studies that did not report standard deviations (SD), we have calculated the SD using the following formula: SD = SE × √N (N represents the group size) (Altman and Bland, 2005). Several independent groups (e.g., different cell transplantation doses and timings) within the same study were treated as separated datasets and considered independent outcomes. In cases where multiple follow-up points were available, only data from the longest follow-up time were extracted.

### 2.5 Quality assessment

The Collaborative Approach to Meta-Analysis and Review of Animal Data from Experimental Studies (CAMARADES) checklist was used to assess each included study (Macleod et al., 2004). The checklist consisted of the following criteria: (1) publication in a peer-reviewed journal; (2) control of temperature; (3) random allocation to treatment or control; (4) allocation concealment; (5) blinded assessment of outcome; (6) avoidance of neuroprotective anesthetics (such as Ketamine); (7) animal model (aged, diabetic, or hypertensive); (8) sample size calculation; (9) compliance with animal welfare regulations; (10) statement of conflict of interest; and (11) pretreatment behavioral assessment. Each of the 11 criteria was scored one point. The assessment was performed independently by two authors, with any disagreements resolved through discussion or consultation with a third author.

### 2.6 Statistical analysis

Statistical analysis was performed using Stata 17.0 (StataCorp, College Station, TX, United States). The primary outcome used for the analysis was the mNSS, while the secondary outcome was lesion volume. Treatment effects were calculated as standardized mean differences (SMD) using a random-effects model and Hedges’ g statistic (Borenstein et al., 2021; Hedges and Olkin, 2014). Heterogeneity among studies was assessed using the *I* ^*2*^ statistic, and when *I* ^*2*^ > 50%, it was considered that there was significant heterogeneity among the studies. A *p*-value of <0.1 was considered statistically significant for heterogeneity. Similarly, a *p*-value of <0.05 was considered statistically significant for differences between the treatment and control groups. To explore sources of heterogeneity, we conducted subgroup analyses and meta-regression. Potential publication bias was evaluated using Egger’s test and visualized with funnel plots (Egger et al., 1997). Additionally, sensitivity analysis was performed by iteratively removing one study at a time.

## 3 Results

### 3.1 Study selection

**FIGURE 1:**
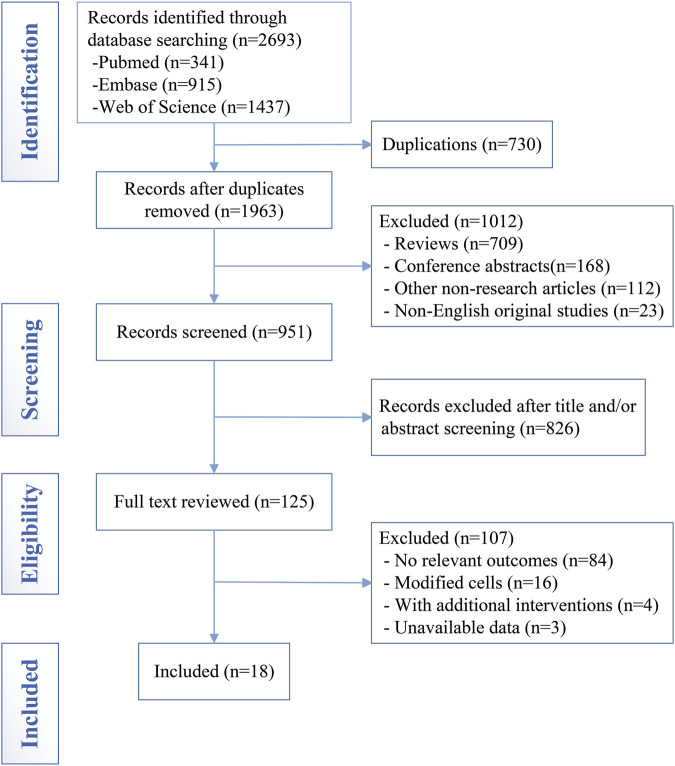
PRISMA flow diagram of included studies for this meta-analysis.

Figure 1 illustrates our screening process and strategy. Using our search methodology, we retrieved a total of 2,693 relevant studies from three databases: 341 from PubMed, 915 from Embase, and 1,437 from Web of Science. After eliminating 730 duplicate publications and 1,012 non-research articles, we selected 951 papers for title/abstract screening. Of these, 125 articles were assessed in full text. Ultimately, 18 studies were included in the meta-analysis (Abedi et al., 2022; Aligholi et al., 2016; Andreu et al., 2023a; Andreu et al., 2023b; Bakshi et al., 2006; Ghandy et al., 2023; Hu et al., 2020; Jahanbazi Jahan-Abad et al., 2018; Lin et al., 2018; Liu et al., 2022; Liu et al., 2014; Narouiepour et al., 2022; Shear et al., 2004; Skardelly et al., 2014; Tao et al., 2017; Wang et al., 2013; Wang et al., 2022; Xue et al., 2015). These 18 studies reported 11 outcomes associated with the mNSS and 18 outcomes associated with lesion volume.

### 3.2 Study characteristics

Table 1 summarizes the key characteristics of the 18 studies (refer to Supplementary Table S1 for more details). Briefly, eight studies originated from China, five from Iran, four from the United States, and one from Germany. Thirteen studies were conducted on rats, while five were conducted on mice. Regarding brain injury models, six studies employed the controlled cortical impact method, five used a biopsy punch, three employed the penetrating traumatic brain injury model, and one study each used the weight drop impact model, fluid percussion injury model, stab wound injury model, and surgical instrument compression. In terms of the stem cell sources, 12 studies employed xenogeneic NSC transplantation, while six used allogeneic sources. Follow-up duration ranged from 14 to 420 days, with a median of 28 days and an interquartile range (IQR) of 28–73.5 days.

**TABLE 1:** Characteristic proportion of 18 included studies.

Characteristics	Summary statistics
Number of studies	18 (100%)
Country	​
China	8 (44.44%)
Iran	5 (27.78%)
United States	4 (22.22%)
Germany	1 (5.56%)
Animal species	
Rat	13 (72.22%)
Mice	5 (27.78%)
Lesion model	
CCI model	6 (33.33%)
Biopsy punch model	5 (27.78%)
pTBI model	3 (16.67%)
WDI model	1 (5.56%)
LFP model	1 (5.56%)
SWI model	1 (5.56%)
Surgical instrument compression model	1 (5.56%)
NSC source	
Allogeneic	6 (33.33%)
Xenogeneic	12 (66.67%)

Percentages may not total 100 due to rounding. CCI, controlled cortical impact; pTBI, penetrating traumatic brain injury; WDI, weight drop impact; LFP, lateral fluid percussion; SWI, stab wound injury; NSC, neural stem cell.

Supplementary Tables S2, S3 summarize the characteristics of outcomes associated with the mNSS and lesion volume, respectively. Among the 18 studies, 11 outcomes were associated with the mNSS. Of these 11 outcomes, ten were associated with NSC transplantation within 1 day of injury, while one was associated with transplantation within 1–7 days. Regarding the transplantation method, 10 outcomes were associated with local transplantation, and one was associated with systemic transplantation via the tail vein. For the transplantation dose, 10 outcomes were associated with a dose of less than 1.0E+06 cells, whereas one with a dose of 1.0E+06 cells or more. Similarly, among the 18 lesion volume outcomes, eight were associated with NSC transplantation within 1 day of injury, eight within 1–7 days, and two after 7 days. All 18 outcomes were associated with local transplantation; fifteen with a transplantation dose of less than 1.0E+06 cells, and three with a dose of 1.0E+06 cells or more.

### 3.3 Quality assessment

The median quality score of the 18 studies was 7.00, with an IQR of 6.25–7.00. Detailed scoring is provided in the CAMARADES checklist (Supplementary Table S4), and the distribution of scores for each item is shown in Table 2. All 18 studies were published in peer-reviewed journals and did not include aged, diabetic, or hypertensive animals. More than half of the studies reported randomized allocation (94.4%), temperature control (72.2%), pretreatment assessment (66.7%), blinded assessment (61.1%), adherence to animal welfare standards (88.9%), and conflict of interest disclosures (66.7%). Additionally, five studies (27.8%) explicitly used non-neuroprotective anesthetics, and three (16.7%) performed *a priori* sample size calculations. None of the studies, however, employed allocation concealment.

**TABLE 2:** Distribution of the quality score meeting with each CAMARADES item.

Item	Number of studies	Percentage
Publication in a peer reviewed journal	18	100.00%
Control of temperature	13	72.2%
Random allocation to treatment or control	17	94.4%
Allocation concealment	0	0.0%
Blinded assessment of outcome	11	61.1%
Avoidance of neuroprotective anesthetics	5	27.8%
Animal model (without aged, diabetic, or hypertensive)	18	100.0%
Sample size calculation	3	16.7%
Compliance with animal welfare regulations	16	88.9%
Statement of conflict of interest	12	66.7%
Pretreatment behavioral assessment	12	66.7%

### 3.4 Effect size

**FIGURE 2:**
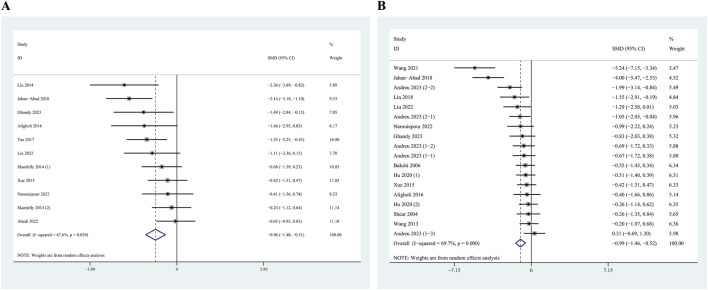
Forest plot of standardized mean difference (SMD) of modified Neurological Severity Score (mNSS) **(A)** and lesion volume **(B)** between neural stem cell (NSC) transplantation group and control group along with a 95% confidence interval (CI).

The pooled effect size for NSC transplantation was calculated using a random-effects model. The results indicated that, in the context of TBI, NSC transplantation led to reductions in both the mNSS and lesion volumes. The meta-analysis of 11 outcomes associated with the mNSS demonstrated a significant difference between the NSC transplantation group and the control group, with an SMD of −0.96 (95% confidence interval [CI]: −1.40 to −0.51, *I* ^*2*^ = 47.6%; Figure 2A). The heterogeneity among these studies was not apparent. Additionally, pooling data from 18 outcomes associated with lesion volume revealed a significant difference, with an SMD of −0.99 (95% CI: −1.46 to −0.52, *I* ^*2*^ = 69.7%; Figure 2B), accompanied by significant heterogeneity. These findings suggest that NSC transplantation exerts a beneficial effect on TBI in rodent models.

### 3.5 Subgroup analysis and meta-regression analysis

To address the heterogeneity observed among the studies, we conducted subgroup analyses (Supplementary Figures S1, S2) and meta-regression (Supplementary Figures S3, S4) to investigate its potential sources. These analyses were categorized based on different animal species, cell sources, transplantation time, transplantation doses, follow-up duration, and TBI models.

The results indicated that NSC transplantation reduced lesion volumes in both rats (SMD = −0.78, 95% CI: −1.21 to −0.35, *I* ^*2*^ = 59.2%) and mice (SMD = −1.96, 95% CI: −3.72 to −0.19, *I* ^*2*^ = 84.9%) (Supplementary Figure S1A). For lesion volume, a statistically significant effect was observed in the xenogeneic subgroup, whereas the allogeneic subgroup showed a similar trend that did not reach statistical significance (Supplementary Figure S1B). This difference may reflect the limited number of studies in the allogeneic subgroup and should not be interpreted as definitive evidence of a true cell source–related difference. Treatments administered within 1 day post-injury (SMD = −1.13, 95% CI: −1.86 to −0.41, *I* ^*2*^ = 68.0%) or within 1–7 days post-injury (SMD = −1.09, 95% CI: −1.84 to −0.33, *I* ^*2*^ = 75.7%) were found to be beneficial, whereas treatments administered beyond 7 days did not yield significant benefits (SMD = −0.18, 95% CI: −1.16 to 0.80, *I* ^*2*^ = 46.8%) (Supplementary Figure S1C). Regarding cell dosage, transplantation of fewer than 1.0E+06 cells (SMD = −1.03, 95% CI: −1.58 to −0.48, *I* ^*2*^ = 72.1%) demonstrated significant benefits, whereas transplantation of 1.0E+06 or more cells (SMD = −0.86, 95% CI: −1.82 to 0.11, *I* ^*2*^ = 66.4%) did not show a statistically significant difference (Supplementary Figure S1D). Significant benefits were observed for follow-up durations of within 1 month (SMD = −1.21, 95% CI: −2.20 to −0.22, *I* ^*2*^ = 75.4%) and 1–3 months (SMD = −1.13, 95% CI: −1.85 to −0.40, *I* ^*2*^ = 75.1%) (Supplementary Figure S1E), whereas follow-up durations beyond 3 months did not show a statistically significant reduction in lesion volume. This long-term subgroup should also be interpreted cautiously because it was based on a limited number of studies. In the subgroup analysis by TBI model, statistically significant effects were observed in several model categories, whereas the lateral fluid percussion (LFP) subgroup did not show a statistically significant effect (SMD = −0.55, 95% CI: −1.43 to 0.34) (Supplementary Figure S1F). However, several model-specific subgroups were represented by only a single study, so the apparent differences between models may reflect study-specific factors rather than true model-related effects.

For the mNSS, NSC transplantation demonstrated benefits in both rats (SMD = −0.91, 95% CI: −1.44 to −0.37, *I* ^*2*^ = 54.8%) and mice (SMD = −1.27, 95% CI: −2.00 to −0.53, *I* ^*2*^ = 0) (Supplementary Figure S2A). Similarly, beneficial effects were observed in both allogeneic and xenogeneic NSC transplantation subgroups (Supplementary Figure S2B), suggesting that the functional improvement associated with NSC transplantation may not be restricted to a single cell source category. Transplantation within 1 day post-injury significantly reduced the mNSS (SMD = −1.03, 95% CI: −1.52 to −0.54, *I* ^*2*^ = 49.7%), while transplantation between 1 and 7 days post-injury (SMD = −0.42, 95% CI: −1.31 to 0.47) showed only one outcome, indicating further research is needed (Supplementary Figure S2C). Regarding cell dosage, transplantation of fewer than 1.0E+06 cells (SMD = −1.06, 95% CI: −1.51 to −0.61, *I* ^*2*^ = 40.4%) was beneficial, whereas transplantation of 1.0E+06 cells or more (SMD = −0.05, 95% CI: −0.93 to 0.83) did not show significant effects (Supplementary Figure S2D). However, this subgroup was based on only one outcome and should therefore be interpreted with considerable caution. For follow-up duration, significant benefits were observed within 1 month (SMD = −1.11, 95% CI: −1.64 to −0.58, *I* ^*2*^ = 50.7%), whereas follow-up periods longer than 1 month did not show a statistically significant difference (SMD = −0.45, 95% CI: −1.08 to 0.18, *I* ^*2*^ = 0) (Supplementary Figure S2E). However, this subgroup was based on only two outcomes, which limits the precision and robustness of the pooled estimate and makes it difficult to determine whether the absence of significance reflects a true lack of longer-term benefit or simply insufficient evidence. For TBI models, significant benefits were observed across all groups (Supplementary Figure S2F). More details have been provided in Table 3.

**TABLE 3:** Subgroup analysis of modified Neurological Severity Score and lesion volume.

Outcome	Group	Subgroup	No of outcome	Heterogeneity		SMD(95%CI)	*p*
				*I* ^2^ (%)	*p*		
mNSS	Animal species	Mice	2	0.0	0.757	−1.27 (−2.00, −0.53)	0.001
		Rats	9	54.8	0.024	−0.91 (−1.44, −0.37)	0.001
	Cell sources	Allogeneic	5	24.8	0.256	−1.18 (−1.77, −0.59)	<0.001
		Xenogeneic	6	58.7	0.033	−0.78 (−1.42, −0.14)	0.017
	Transplantation time	≤1 d	10	49.7	0.036	−1.03 (−1.52, −0.54)	<0.001
		>1d, ≤ 7 d	1	-	-	−0.42 (−1.31, 0.47)	0.355
	Transplantation doses	<1.0E+06	10	40.4	0.088	−1.06 (−1.51, −0.61)	<0.001
		≥1.0E+06	1	-	-	−0.05 (−0.93, 0.83)	0.912
	Duration	≤1 m	9	50.7	0.039	−1.11 (−1.64, −0.58)	<0.001
		>1 m, ≤ 3 m	2	0	0.494	−0.45 (−1.08, 0.18)	0.162
	TBI models	WDI	1	-	-	−2.36 (−3.89, −0.82)	0.003
		Biopsy punch	5	63.6	0.027	−1.06 (−1.92, −0.21)	0.015
		SWI	1	-	-	−1.35 (−2.25, −0.45)	0.003
		CCI	4	0	0.705	−0.54 (−1.01, −0.06)	0.027
Lesion volume	Animal species	Mice	4	84.9	<0.001	−1.96 (−3.72, −0.19)	0.030
		Rats	14	59.2	0.003	−0.78 (−1.21, −0.35)	<0.001
	Cell sources	Allogeneic	4	86.2	<0.001	−1.68 (−3.39, 0.03)	0.054
		Xenogeneic	14	61.0	0.002	−0.85 (−1.30, −0.39)	<0.001
	Transplantation time	≤1 d	8	68.0	0.003	−1.13 (−1.86, −0.41)	0.002
		>1d, ≤ 7 d	8	75.7	<0.001	−1.09 (−1.84, −0.33)	0.005
		>7 d	2	46.8	0.170	−0.18 (−1.16, 0.80)	0.713
	Transplantation doses	<1.0E+06	15	72.1	<0.001	−1.03 (−1.58, −0.48)	<0.001
		≥1.0E+06	3	66.4	0.051	−0.86 (−1.82, 0.11)	0.082
	Duration	≤1 m	6	75.4	0.001	−1.21 (−2.20, −0.22)	0.016
		>1 m, ≤ 3 m	9	75.1	<0.001	−1.13 (−1.85, −0.40)	0.002
		>3 m	3	0	0.912	−0.35 (−0.90, 0.19)	0.206
	TBI models	Biopsy punch	4	80.9	0.001	−1.51 (−2.98, −0.04)	0.044
		CCI	5	83.9	<0.001	−1.27 (−2.53, −0.01)	0.049
		pTBI	7	41.3	0.115	−0.65 (−1.14, −0.16)	0.009
		Surgical instrument compression	1	—	—	−1.55 (−2.91, −0.19)	0.026
		LFP	1	—	—	−0.55 (−1.43, 0.34)	0.226

mNSS, modified Neurological Severity Score; TBI, traumatic brain injury; WDI, weight drop impact; SWI, stab wound injury; CCI, controlled cortical impact; pTBI, penetrating traumatic brain injury; LFP, lateral fluid percussion.

The univariate meta-regression analysis for both the mNSS and lesion volume outcomes showed no significant associations between the examined variables (animal species, cell sources, transplantation time, transplantation dose, follow-up duration, and TBI models) and the SMD (Supplementary Figures S3, S4). None of the variables were statistically significant (*p*-values ranging from 0.150 to 0.835; further details provided in Table 4).

**TABLE 4:** Meta regression of modified Neurological Severity Score and lesion volume.

Outcome	Variables	Coefficient	95% CI	Std. Err	*p*
mNSS	Animal species	0.35	(−1.02, 1.72)	0.61	0.575
	Cell source	0.45	(−0.61, 1.51)	0.47	0.365
	Transplantation time	0.61	(−1.10, 2.32)	0.75	0.439
	Transplantation dose	1.01	(−0.53, 2.56)	0.68	0.171
	Duration	0.64	(−0.60, 1.89)	0.55	0.271
	TBI models	0.34	(−0.15, 0.82)	0.21	0.150
Lesion volume	Animal species	0.99	(−0.53, 2.52)	0.72	0.185
	Cell source	0.65	(−0.90, 2.21)	0.74	0.387
	Transplantation time	0.34	(−0.59, 1.28)	0.44	0.448
	Transplantation dose	0.17	(−1.52, 1.86)	0.80	0.835
	Duration	0.38	(−0.53, 1.28)	0.43	0.389
	TBI models	0.24	(−0.35, 0.83)	0.28	0.405

mNSS, modified Neurological Severity Score; TBI, traumatic brain injury.

It should be noted that neither subgroup analysis nor meta-regression identified sources of heterogeneity with statistical significance. However, the analysis of lesion volume revealed a significant reduction in heterogeneity within the subgroups of ‘Rats,’ ‘Xenogeneic,’ ‘> 7 d,’ ‘> 3 m,’ and ‘pTBI’ models. On the other hand, for the mNSS, a significant decrease in heterogeneity was observed in the subgroups of ‘Mice,’ ‘Allogeneic,’ ‘< 1.0E+06,’ ‘> 1 m, ≤ 3 m,’ and ‘CCI’ models. These findings indicate that the meta-analysis results are more reliable within these specific subgroups and that this classification method may account for part of the observed heterogeneity. Nevertheless, other factors may contribute to the sources of heterogeneity.

### 3.6 Publication bias

**FIGURE 3:**
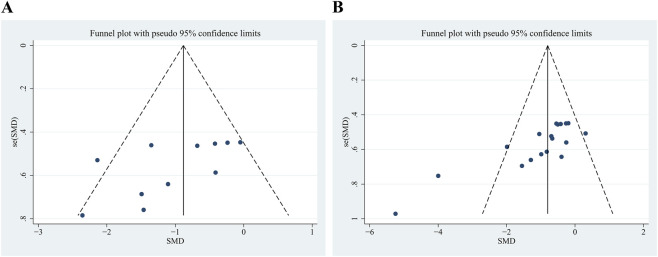
Funnel plot for modified Neurological Severity Score (mNSS) **(A)** and lesion volume **(B)**.

To assess the potential for publication bias, we generated funnel plots and conducted Egger’s test. The funnel plot for the mNSS appeared largely symmetrical around the effect size axis, indicating a lower risk of publication bias (Figure 3A). However, the funnel plot for lesion volume exhibited asymmetry, suggesting a potential publication bias in these studies (Figure 3B). These findings should be interpreted with caution within the broader context of the meta-analysis. Egger’s test for the mNSS outcome indicated no significant evidence of publication bias (*p* = 0.051) (Supplementary Figure S5A). Conversely, the test for lesion volume revealed significant evidence of publication bias (*p* < 0.001) (Supplementary Figure S5B). Therefore, trim-and-fill analysis was performed. However, no studies were trimmed or imputed, indicating that the pooled estimate remained unchanged after trim-and-fill adjustment.

### 3.7 Sensitivity analyses

**FIGURE 4:**
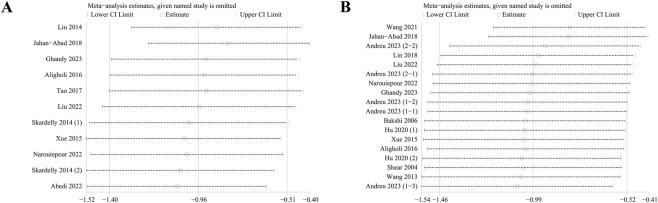
Sensitivity analysis of the studies included in modified Neurological Severity Score (mNSS) **(A)** and lesion volume outcomes **(B)**.

Sensitivity analyses were conducted to assess the robustness of the meta-analytic results (Figures 4A,B). For both outcomes, an influence analysis showed that excluding any single study did not result in significant changes to the pooled SMD, indicating that the findings are robust and stable.

## 4 Discussion

To the best of our knowledge, this is the first meta-analysis to evaluate the effectiveness of NSC transplantation in rodent models of TBI. The findings indicate that NSC transplantation effectively reduces both the mNSS and lesion volumes, providing robust evidence for the efficacy of this intervention.

Although the number of studies included is limited, these results are consistent with previous systematic reviews in related fields. For example, Peng et al. conducted a meta-analysis of 24 studies on the efficacy of mesenchymal stem cells (MSCs) in animal models of TBI, finding beneficial effects on motor function recovery (Peng et al., 2015). Similarly, Yang et al.'s network meta-analysis of 60 studies demonstrated that MSC-derived extracellular vesicles might facilitate motor function recovery in models of TBI or spinal cord injury (Yang et al., 2023). Although these studies focused on MSCs—likely owing to more established collection techniques and earlier research—they indirectly support our findings that neural-derived stem cells, such as NSCs, may also be effective in treating neurological disorders.

Subsequent subgroup analysis revealed that NSC transplantation consistently provides significant benefits in reducing lesion volume and the mNSS, irrespective of the animal species. This finding is consistent with meta-analyses of other stem cell therapies and animal disease models (Zhao et al., 2023; He et al., 2021). However, transplants performed more than 7 days post-injury, as well as those using doses of 1.0E+06 cells or more, did not show statistically significant effects in the present subgroup analyses. Importantly, the lesion volume subgroup for transplantation beyond 7 days was represented by only two datasets derived from a single study, although these datasets corresponded to independent intervention arms. Therefore, this finding should not be interpreted as robust evidence against delayed NSC transplantation, but rather as insufficient evidence to draw firm conclusions regarding late intervention timing. Results from other studies still suggest potential trends. For instance, Shear and colleagues identified the optimal timing for NSC transplantation between 2 and 7 days post-injury (Shear et al., 2011). Similarly, Shang’s network meta-analysis on spinal cord injury highlighted that the best outcomes were observed during the subacute phase (3–7 days, including day 7 but excluding day 3) (Shang et al., 2022). These differences can be explained by the dynamic nature of the injury microenvironment. In the acute and subacute phases, increased neuroinflammation, glial activation, and tissue repair mechanisms may foster an environment conducive to NSC survival, integration, and paracrine effects. In contrast, delayed transplantation may coincide with glial scar formation, creating a more inhibitory microenvironment that limits the therapeutic potential of NSCs (Nicaise et al., 2021; Nishimura et al., 2013). With advances in transcriptomic and sequencing technologies, it has become possible to identify changes at various stages of injury more accurately, aiding in the determination of the optimal transplantation time window (Ding et al., 2022). Future research is expected to focus on determining the optimal transplantation timing and understanding the molecular changes occurring at different stages.

Regarding transplantation dose, Yousefifard et al. conducted a meta-analysis and found that the optimal number of adipose-derived stem cells for animal models of ischemic stroke is 1.0E+06 cells. However, this finding requires further validation (Yousefifard et al., 2020). In contrast, current analysis indicated that transplantation of 1.0E+06 or more cells did not demonstrate a statistically significant benefit compared with transplantation of fewer than 1.0E+06 cells. This finding should be interpreted cautiously, as the ≥1.0E+06 subgroup was based on only a small number of datasets and the included studies differed in important design features, such as transplantation timing, follow-up duration, disease model, and other experimental conditions. Therefore, the apparent lack of benefit in the higher-dose subgroup cannot be attributed to dose alone. Higher cellular dosages may reduce cell survival due to limited space and resources within the host tissue, resulting in overcrowding, competition for oxygen and nutrients, and heightened immune responses. Conversely, excessively low dosages may fail to provide sufficient therapeutic effects, as the transplanted cells may not be present in adequate numbers to exert neuroprotective or reparative functions (Sondermeijer et al., 2018). Therefore, optimizing the cell dosage is critical to balancing efficacy and safety. Future dose-dependent studies, coupled with advanced imaging and tracking techniques, are needed to determine the optimal transplantation range and clarify the mechanisms by which dosage influences cell survival, integration, and functional recovery.

A relatively high degree of heterogeneity was observed for the lesion volume outcome (*I* ^*2*^ = 69.7%). In the context of preclinical TBI research, such heterogeneity is likely multifactorial and may reflect both biological and methodological diversity across studies. The included experiments differed substantially in animal species, injury paradigms, transplantation timing, cell dose, follow-up duration, and cell source. These differences are unlikely to be trivial, because each factor may influence the host inflammatory milieu, graft survival, tissue remodeling, and the trajectory of structural recovery after TBI.

In particular, heterogeneity in lesion volume may be expected to exceed that of functional outcomes because structural endpoints are highly sensitive to differences in injury model and assessment protocol. The included studies employed multiple TBI models, including CCI, LFP, WDI, SWI, biopsy punch, and pTBI, which represent distinct injury mechanisms and may produce different patterns of tissue loss and cavitation. In addition, lesion volume is influenced by the timing of assessment, since tissue damage evolves dynamically from the acute phase to chronic tissue remodeling. Differences in lesion quantification methods, histological processing, and follow-up duration may therefore have contributed further to between-study variability.

Although we performed subgroup analyses and univariate meta-regression, none of the examined variables showed statistically significant associations with effect size. This lack of statistical significance should not be interpreted as evidence that these factors are unimportant. Rather, it may reflect the limited statistical power of exploratory heterogeneity analyses in a meta-analysis with a relatively small number of studies and uneven subgroup distribution. For example, some lesion volume subgroup categories contained only a small number of datasets, which reduces the ability of both subgroup analysis and meta-regression to detect true effect modification. Accordingly, the current analyses are better viewed as exploratory rather than confirmatory.

Notably, heterogeneity decreased in several lesion volume subgroups, such as rats, xenogeneic transplantation, >7 days, >3 months, and pTBI models. While these observations may suggest that part of the heterogeneity is related to experimental design, they should be interpreted cautiously and not be overgeneralized. Overall, the pooled estimate for lesion volume should be understood as an average effect across diverse preclinical settings rather than a precise estimate for any single model or transplantation protocol. Future preclinical studies using more standardized injury paradigms, transplantation regimens, outcome definitions, and reporting practices will be essential to improve comparability and strengthen the interpretability of evidence synthesis in this field.

An additional point that merits attention is the effect of follow-up duration on lesion volume outcomes. In the present subgroup analysis, significant reductions in lesion volume were observed at follow-up durations within 1 month and within 1–3 months, whereas the subgroup with follow-up beyond 3 months did not show a statistically significant benefit. This finding may suggest that the structural benefits of NSC transplantation are more apparent in the short to intermediate term, while the persistence of these effects over longer periods remains uncertain. However, the number of studies with longer follow-up was small, and these studies differed in injury model, transplantation timing, and other experimental conditions. Therefore, the current evidence is insufficient to determine whether NSC transplantation provides sustained long-term benefits on lesion volume after TBI.

Moreover, we reviewed the safety events reported in the included studies. Although few studies explicitly reported severe systemic adverse events, several raised potential concerns. For instance, Skardelly et al. reported delayed microglial activation and macrophage infiltration at 12 weeks post-transplantation, suggesting the potential for chronic inflammatory responses (Skardelly et al., 2014). Furthermore, Xue et al. (2015) reported increased blood-brain barrier permeability and vascular leakage at the transplantation site, underscoring the need for monitoring local microenvironmental changes. At the same time, clinical trials targeting chronic spinal cord injury and progressive multiple sclerosis have demonstrated the safety of NSC transplantation. However, these studies still emphasize the need for long-term monitoring to evaluate chronic risks and optimize patient selection (Genchi et al., 2023; Curtis et al., 2018). Collectively, these findings suggest that, in addition to optimizing transplantation protocols—such as dosage, timing, and TBI models—there is a critical need for long-term, large-scale preclinical trials to comprehensively assess chronic risks, which are essential for the clinical translation of NSC therapies.

Another important issue is the potential for publication bias in the lesion volume outcome. The asymmetric funnel plot and significant Egger’s test (*p* < 0.001) raise concern about small-study effects and/or selective publication. In preclinical research, smaller studies may be more likely to report larger treatment effects, whereas studies with null or negative findings may remain unpublished or be more difficult to identify through conventional database searches. As a result, the pooled effect size for lesion volume may have been overestimated in the present meta-analysis. To further assess this issue, we performed an exploratory trim-and-fill analysis for lesion volume. No studies were imputed and the pooled estimate remained unchanged. This result does not exclude the possibility of bias; rather, it suggests that the observed funnel plot asymmetry may not be fully explained by the specific missing-study mechanism assumed by the trim-and-fill method. In the presence of substantial heterogeneity and possible outlying small studies, trim-and-fill results should themselves be interpreted cautiously. Future preclinical studies should emphasize prospective registration, transparent reporting, and publication of negative as well as positive findings to reduce the risk of publication bias and improve the reliability of evidence synthesis.

Meanwhile, some limitations should be noted in this current study. Firstly, we included only English-language original research, which may limit the comprehensiveness of the literature and introduce language bias. Secondly, our search was confined to PubMed, Embase, and Web of Science, potentially excluding relevant studies from specialized databases. Thirdly, we did not include gray literature, which may bring publication bias. Indeed, funnel plot analysis and Egger’s test suggest potential publication bias, particularly for lesion volume outcomes, indicating that studies with insignificant results may have been underrepresented. As a result, the observed effects may be overestimated. In addition, the total number of included studies was relatively limited, and several subgroup analyses were based on sparse datasets, particularly the >7 days lesion-volume subgroup, the ≥1.0E+06 mNSS subgroup, the mNSS subgroup with follow-up >1 month, the lesion-volume subgroup with follow-up >3 months, and several TBI model-specific subgroups represented by only a single study. Therefore, these subgroup findings should be considered exploratory and interpreted with caution. Some datasets were also derived from multiple intervention arms within the same study, which may have reduced the independence of subgroup comparisons. Despite these limitations, the present findings provide supportive preclinical evidence for the potential therapeutic value of NSC transplantation after TBI and highlight the need for larger, standardized, and longer-term preclinical studies.

## 5 Conclusion

NSC transplantation has been shown to reduce both the mNSS and lesion volume in rodent models of TBI, suggesting its potential as a promising treatment for patients with TBI in the future. However, several challenges remain in preclinical research. For example, long-term safety data are still needed, along with more consistent protocols. A clearer understanding of the mechanisms underlying functional recovery is also necessary. To facilitate successful clinical translation, it is essential to define optimal transplantation parameters—such as cell dose, timing, and delivery route—and to establish standardized outcome measures that closely mirror clinical endpoints and ensure reproducibility. Furthermore, reducing publication bias by encouraging the registration of preclinical studies and the publication of negative results is also crucial. Ultimately, well-designed early-phase clinical trials, guided by robust preclinical evidence and focusing on both efficacy and safety, will be critical in determining whether NSC transplantation can fulfill its therapeutic potential for patients with TBI.

## Data Availability

The original contributions presented in the study are included in the article/Supplementary Material, further inquiries can be directed to the corresponding author.
