# Supplementary material for: Neural stem cell transplantation in rodent models of traumatic brain injury: a systematic review and meta-analysis
Source: Front Bioeng Biotechnol. 2026 May 21;14:1823886. doi: 10.3389/fbioe.2026.1823886 (PMC13233701; doi:10.3389/fbioe.2026.1823886)
Supplement: Supplementary file 1 [file Supplementaryfile1.docx]

Supplementary Material

# Supplementary Data

None.

# Supplementary Figures and Tables

For more information on Supplementary Material and for details on the different file types accepted, please see [here](https://www.frontiersin.org/guidelines/author-guidelines#supplementary-material).

## Supplementary Figures





**Supplementary Fig. 1** Forest plot of standardized mean difference (SMD) of lesion volume between neural stem cell (NSC) transplantation group and control group along with a 95% confidence interval (CI) across different animal species (A), cell sources (B), transplantation time (C), transplantation doses (D), follow-up duration (E) and traumatic brain injury (TBI) models (F).



**Supplementary Fig. 2** Forest plot of standardized mean difference (SMD) of modified Neurological Severity Score (mNSS) between neural stem cell (NSC) transplantation group and control group along with a 95% confidence interval (CI) across different animal species (A), cell sources (B), transplantation time (C), transplantation doses (D), follow-up duration (E) and traumatic brain injury (TBI) models (F).



**Supplementary Fig. 3** Meta-regression analysis for related variables and the standardized mean difference (SMD) of modified Neurological Severity Score (mNSS). Animal species (A), cell source (B), transplantation time (C), transplantation dose (D), follow-up duration (E) and traumatic brain injury (TBI) models (F).



**Supplementary Fig. 4** Meta-regression analysis for related variables and the standardized mean difference (SMD) of lesion volume. Animal species (A), cell source (B), transplantation time (C), transplantation dose (D), follow-up duration (E) and traumatic brain injury (TBI) models (F).
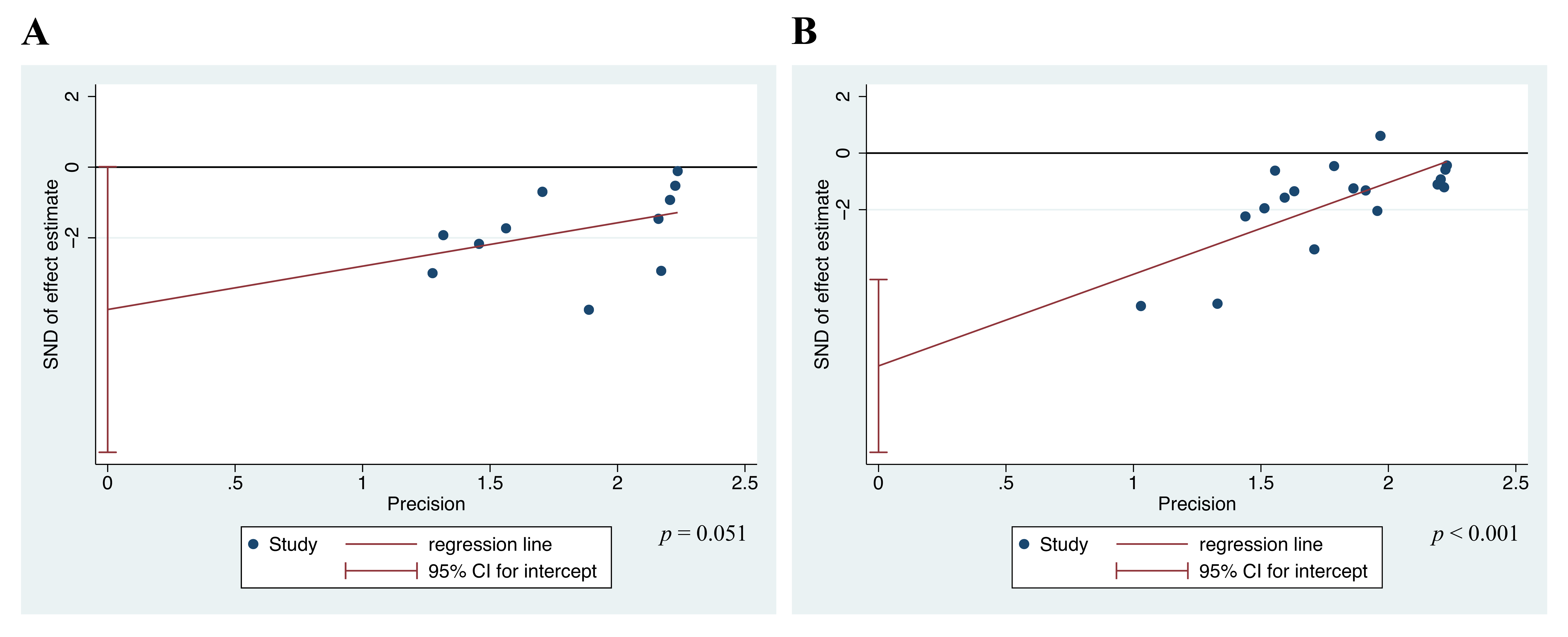


**Supplementary Fig. 5** Egger's test of the studies included in modified Neurological Severity Score (mNSS) (A) and lesion volume outcomes (B).
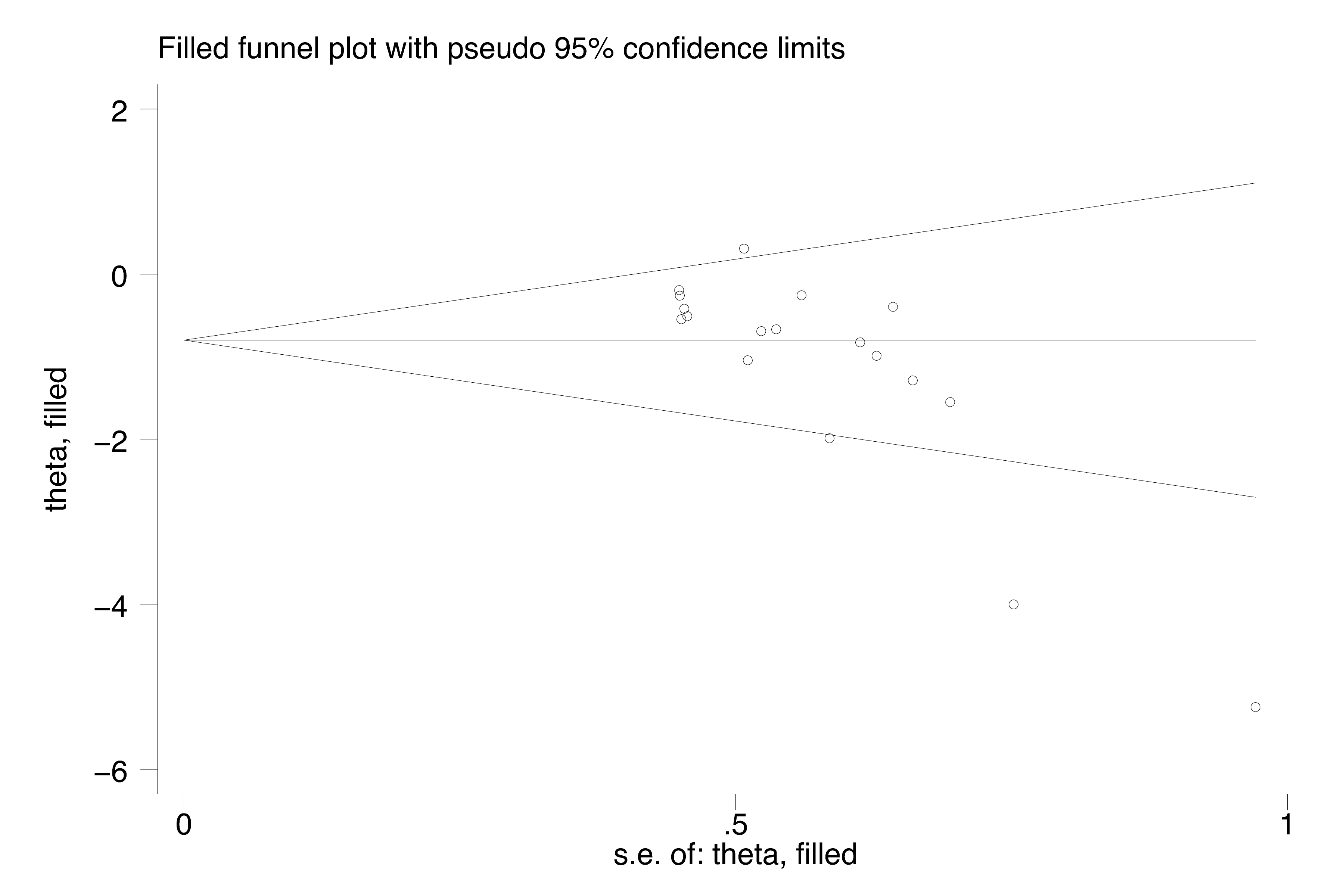


**Supplementary Fig. 6** Trim-and-fill method was used to evaluate the missing studies in lesion volume outcomes.

## Supplementary Tables

**Supplementary Table 1.** Characteristics of the 18 included studies.

| Number | Study | Animal | | | TBI Model | NSC transplantation | | | | Duration |
| --- | --- | --- | --- | --- | --- | --- | --- | --- | --- | --- |
|  |  | Species | Sex | Weight |  | Source | Dose  (cells) | Route | Time(post-injury) |  |
| 1 | Shear, 2004, USA | C57BL.6J mice | Male | - | CCI | Xenogeneic | 1.0E+05 | Local | 7 days | 420 days |
| 2 | Bakshi, 2006, USA | SD rat | Male | 350-400 g | LFP | Xenogeneic | 1.0E+05 | Local | ≤ 1 day | 42 days |
| 3 | Wang, 2013, China | SD rat | Male | 280-320g | CCI | Xenogeneic | 5.0E+05 | Local | ≤ 1 day | 28 days |
| 4 | Skardelly, 2014, Germany | SD rat | Male | 280-320 g | CCI | Xenogeneic | 1.0E+05/5.0E+05 | Local/ Systemic | ≤ 1 day | 84 days |
| 5 | Liu, 2014, China | SD rat | Female | 200 ± 20 g | WDI | Allogeneic | 2.0E+05 | Local | ≤ 1 day | 14 days |
| 6 | Xue, 2015, China | Wistar rat | Female | 250-280 g | CCI | Allogeneic | 1.0E+05 | Local | 7 days | 28 days |
| 7 | Aligholi, 2016, Iran | Wistar rat | Male | 230-250g | Biopsy punch | Allogeneic | 5.0E+05 | Local | ≤ 1 day | 28 days |
| 8 | Tao, 2017, China | C57BL/6J mice | Male | 30-35 g | SWI | Allogeneic | 1.0E+04 | Local | ≤ 1 day | 14 days |
| 9 | Jahan-Abad, 2018, Iran | Wistar rat | Male | 220-250 g | Biopsy punch | Xenogeneic | 5.0E+05 | Local | ≤ 1 day | 28 days |
| 10 | Lin, 2018, China | C57BL/6 mice | - | - | Surgical instrument compression | Xenogeneic | 1.0E+05 | Local | ≤ 1 day | 35 days |
| 11 | Hu, 2020, China | SD rat | Male | ≥ 280 g | pTBI | Xenogeneic | 1.0E+06 | Local | 7 days | 91 days |
| 12 | Wang, 2021, China | C57BL/6 mice | Male | 20-25 g | CCI | Allogeneic | 3.0E+05 | Local | 3 days | 35 days |
| 13 | Liu, 2022, China | C57BL/6 mice | Male | 22-25 g | CCI | Allogeneic | 2.0E+05 | Local | ≤ 1 day | 21 days |
| 14 | Abedi, 2022, Iran | Wistar rat | Male | 220-250 g | Biopsy punch | Xenogeneic | 2.0E+06 | Local | ≤ 1 day | 28 days |
| 15 | Narouiepour, 2022, Iran | Wistar rat | Male | 200 ± 20 g | Biopsy punch | Xenogeneic | 5.0E+05 | Local | ≤ 1 day | 28 days |
| 16 | Andreu, 2023, USA (1) | SD rat | Male | ≤ 280 g | pTBI | Xenogeneic | 1.0E+05 | Local | 7/14/28 days | 84 days |
| 17 | Andreu, 2023, USA (2) | SD rat | Male | ~ 280 g | pTBI | Xenogeneic | 1.6E+05/1.6E+06 | Local | 7 days | 90 days |
| 18 | Ghandy, 2023, Iran | Wistar rat | Male | 200 ± 20 g | Biopsy punch | Xenogeneic | 6.0E+05 | Local | ≤ 1 day | 28 days |

Note: SD rat, Sprague-Dawley rat; TBI, traumatic brain injury; NSC, neural stem cell; CCI, controlled cortical impact; LFP, lateral fluid percussion; WDI, weight drop impact; SWI, stab wound injury; pTBI, penetrating traumatic brain injury.

**Supplementary Table 2.** Characteristic proportion of 11 outcomes of modified Neurological Severity Score.

| Characteristics | Summary statistics |
| --- | --- |
| Number of outcomes | 11 (100%) |
| Transplantation time (post-injury) |  |
| ≤ 1d | 10 (90.91%) |
| >1d, ≤ 7d | 1 (9.09%) |
| > 7d | 0 (0) |
| Transplantation site  Local  Systemic  Transplantation dose  < 1.0E+06  ≥ 1.0E+06 | 10 (90.91%)  1 (9.09%)  10 (90.91%)  1 (9.09%) |

Note: Percentages may not total 100 due to rounding.

**Supplementary Table 3.** Characteristic proportion of 18 outcomes of lesion volume.

| Characteristics | Summary statistics |
| --- | --- |
| Number of outcomes | 18 (100%) |
| Transplantation time (post-injury) |  |
| ≤ 1d | 8 (44.44%) |
| >1d, ≤ 7d | 8 (44.44%) |
| > 7d | 2 (11.11%) |
| Transplantation site  Local  Systemic  Transplantation dose  < 1.0E+06  ≥ 1.0E+06 | 18 (100%)  0 (0)  15 (83.33%)  3 (16.67%) |

Note: Percentages may not total 100 due to rounding.

**Supplementary Table 4.** CAMARADES checklists of 18 reviewed studies

| **Study ID** | **Publication in a peer reviewed journal** | **Control of temperature** | **Random allocation to treatment or control** | **Allocation concealment** | **Blinded assessment of outcome** | **Avoidance of neuroprotective anesthetics** | **Animal model (without aged, diabetic, or hypertensive)** | **Sample size calculation** | **Compliance with animal welfare regulations** | **Statement of conflict of interest** | **Pretreatment behavioral assessment** | **Score** |
| --- | --- | --- | --- | --- | --- | --- | --- | --- | --- | --- | --- | --- |
| Shear, 2004 | Y | N | Y | N | N | N | Y | N | Y | N | Y | 5 |
| Bakshi, 2006 | Y | Y | Y | N | Y | Y | Y | N | Y | N | N | 7 |
| Wang, 2013 | Y | Y | Y | N | Y | N | Y | N | Y | N | Y | 7 |
| Liu, 2014 | Y | Y | Y | N | Y | Y | Y | N | Y | Y | Y | 9 |
| Skardelly, 2014 | Y | Y | Y | N | Y | N | Y | N | N | Y | Y | 7 |
| Xue, 2015 | Y | Y | Y | N | N | Y | Y | N | Y | Y | Y | 8 |
| Aligholi, 2016 | Y | N | Y | N | Y | N | Y | N | Y | N | Y | 6 |
| Tao, 2017 | Y | Y | Y | N | N | Y | Y | N | Y | Y | N | 7 |
| Lin, 2018 | Y | Y | Y | N | N | Y | Y | N | Y | N | Y | 7 |
| Jahan-Abad, 2018 | Y | Y | Y | N | Y | N | Y | N | N | Y | Y | 7 |
| Hu, 2020 | Y | N | Y | N | Y | N | Y | Y | Y | Y | N | 7 |
| Wang, 2021 | Y | Y | Y | N | Y | N | Y | N | Y | Y | N | 7 |
| Liu, 2022 | Y | N | Y | N | Y | N | Y | N | Y | N | Y | 6 |
| Abedi, 2022 | Y | Y | Y | N | N | N | Y | N | Y | Y | N | 6 |
| Narouiepour, 2022 | Y | Y | Y | N | Y | N | Y | N | Y | Y | Y | 8 |
| Ghandy, 2023 | Y | Y | N | N | N | N | Y | N | Y | Y | Y | 6 |
| Andreu, 2023 (1) | Y | N | Y | N | Y | N | Y | Y | Y | Y | Y | 8 |
| Andreu, 2023 (2) | Y | Y | Y | N | N | N | Y | Y | Y | Y | N | 7 |
| Total | 18 | 13 | 17 | 0 | 11 | 5 | 18 | 3 | 16 | 12 | 12 |  |
| Percentage | 100.0% | 72.2% | 94.4% | 0.0% | 61.1% | 27.8% | 100.0% | 16.7% | 88.9% | 66.7% | 66.7% |  |
